# An Account of the Good Effects of Opium Administered in Clysters, in Cases of Menorrhagia

**Published:** 1793

**Authors:** Peter Copland

**Affiliations:** Surgeon at Swayfield, Near Colsterworth, in Lincolnshire.


IX. *An Account of the good Effects of Opium, adminiſtered in Clyſters, in Caſes of Menorrhagia. By Mr.* Peter Copland, *Surgeon at Swayfield, near Colſterworth, in Lincolnſhire.*

### CASE I.

A woman at Colſterworth aged thirty-two years, and who had had four children, was attacked tacked on the third of Auguſt, 1787, with a diſcharge of blood from the uterus, accompanied with pains in the loins and lower part of the abdomen. From the diſappearance of the menſes ſhe ſuppoſed herſelf in the third month of pregnancy; and her midwife, who was ſent for, concluded from ſome ſubſtances expelled with the diſcharge, that abortion had taken place: but the hæmorrhage not ceaſing, I was called to her aſſiſtance the next day. From this time till the eighth of November, bleeding, nitre, acids, alum, bark, and opiates, were uſed by turns, or conjointly, as circumſtances ſeemed to indicate; but with temporary relief only, the pain and hæmorrhage always returning, and, at times, hardly yielding to the means employed.

The patient was now much reduced; and, as I ſuſpected that there was a local diſeaſe of the uterus, I was apprehenſive that the caſe would terminate unfavourably. It, however, at this time occurred to me that Dr. Whytt, in his Obſervations on Nervous Diſorders, had noticed the ſubject of uterine hæmorrhages; and accordingly, on conſulting that work, I found the following remark: " When a pro-" fluvium menſium, or a flooding after abor-

" tion, is attended with, or preceded by, an acute
" pain, not inflammatory, in the lower part of
" the back or belly, and returns with greater
" violence; as often as the pain returns or in-
" creafes, opium will prove a more effectual
" remedy than any of the aftringents."—I therefore, in imitation of the treatment ufed in the cafe there defcribed *, directed that fifty drops of tincture of opium fhould be mixed with a large tea cupful of cold water, and adminiftered every night as a clyfter; and that coftivenefs fhould be obviated by the occafional ufe of laxative clyfters. The reduced ftate of the patient, from the long continuance of the hæmorrhage, induced me to continue the ufe of aftringents alfo in the day time.

From the firft employment of the anodyne clyfter fhe experienced much relief. It was repeated at firft every night, then every fecond night, and afterwards occafionally till the twenty-feventh of November, when fhe was quite well.

Being much ftruck with the alteration that fucceeded the adminiftration of the anodyne clyfter in this cafe, I determined to give it a

* See the edition of his works, in 4to. p. 661.

further further trial on future occaſions. The following caſes have ſince occurred.—I ſhall relate them from the notes I took of each.

### CASE II.

A woman at Caſtle-Bytherm, aged forty-four years, was attacked on the thirtieth of September, 1791, with a diarrhœa, unaccompanied with teneſmus; ſoon after which the menſtrual diſcharge took place (a fortnight only after its laſt appearance) with pain, returning at intervals, and extending from the upper part of the right groin acroſs the abdomen, and ſometimes acroſs the loins. Theſe ſymptoms continued on the fourth of October, when I firſt ſaw her. Her pulſe was then ſmall and quick. A ſolution of magneſia vitriolata in water was directed to be taken immediately, and an anodyne aſtringent draught after ſufficient evacuations.

5th. She had ſeveral evacuations yeſterday, and a good night; but two looſe ſtools this morning.—An aſtringent mixture, with tincture of opium, was directed to be taken at intervals.

6th. The purging ceafed yefterday; but the uterus, with the pain of the abdomen, ftill continued unabated. Sixty drops of tincture of opium in five ounces of water were directed to be injected *per anum*.

7th. The difcharge was leffened, but the pain continued in a confiderable degree. As fhe had had no ftool fince the 5th in the morning, a rhubarb bolus was ordered to be taken immediately. This produced an evacuation, and the clyfter with opium was then repeated. A piece of folded linen, wetted with tincture of opium, was applied to the right groin* after the adminiftration of the clyfter.

11th. The pain ceafed, and the difcharge difappeared after the laft clyfter; but the purging having returned, recourfe was again had to the aftringent mixture. I did not fee her again till the firft of November following, when fhe informed me that the purging ftopped after the repetition of the mixture, and that fhe had continued well ever fince.

* Upon examination no appearance of rupture could be difcovered.

### CASE III.

In my way through Creeton, on the nineteenth of October, 1791, I was defired to fee a woman aged forty-two years, the mother of feveral children, who had mifcarried after having gone above half her time, on the eleventh inftant. From that day fhe had had a coloured difcharge, in more or lefs degree, from the uterus, till a few hours before I faw her, when fhe had been feized with a fhivering, followed by periodic pains in the loins and abdomen, and an alarming increafe of the difcharge, with frequent faintings.

I found her on the bed with her cloaths on, and her face befet with cold fweat. Upon taking hold of her arm, with the intention of examining the ftate of her pulfe, it felt cold and moiftened my fingers with its fweat, which fhe faid extended to every part of her; and fhe repeatedly complained that her feet were very cold. Her pulfe was fmall and irregular, beating one hundred and thirty times in a minute, and difappearing upon the leaft preffure from my fingers. A clyfter, compofed of feventy drops of tinctincture of opium, and a large tea cupful of water, was immediately injected. She was desired not to undergo the fatigue of undressing but to remain upon the bed, to take strong broth cool frequently, and occasionally small quantities of red wine mixed with water, if the discharge would permit. The room was directed to be kept well aired and cool, and cloths were introduced within the os externum, with a view to impede the discharge.

20th. The pains had diminished after the introduction of the clyster: she had got some sleep during the night, and the discharge had been inconsiderable. The universal cold sweat was still present, and vomiting took place this morning. Her pulse was nearly the same as yesterday. The clyster with opium was directed to be repeated in the evening, or at any time in the day, should the pain or discharge require it. Four table spoonfuls of a mixture composed of a pint of decoction of Peruvian bark, and two drachms of acid elixir of vitriol, were ordered to be taken every three hours.

21st. The clyster had been administered yesterday evening; she had had a good night, and the sweating and discharge had disappeared. She retched once this morning. The exertion this occasioned occafioned forced out a large coagulum of blood with the cloths from the vagina. Her pulfe was 110, regular, and lefs feeble. No motion having taken place fince the 19th, a laxative clyfter was directed to be now employed; the ufe of the mixture was continued; and the clyfter with opium was repeated in the evening.

The laxative clyfter yefterday produced a flight evacuation of fæces. She had now no pain or difcharge, and her pulfe was at 100. The clyfter with opium was not repeated after this; but fhe occafionally had recourfe to the laxative clyfter, and perfevered in the ufe of the mixture till the tenth of November, at which time fhe had regained her ufual health.

The preceding cafes to me afford a ftrong proof of the juftnefs of Dr. Whytt's obfervation; and, as I apprehend that it has not been fufficiently attended to, I am defirous of offering them to the medical reader, with the view of rendering the practice as general as its importance feems to deferve.

*Swayfield,*

October 16, 1792.

