# Erratum

**Published:** 1794

**Authors:** 


ERRATUM.

In Vol. IV. page 122, line 1, *for* but the uterus, *read* but the difcharge from the uterus.

